# Physicians’ job satisfaction and motivation in a public academic hospital

**DOI:** 10.1186/s12960-016-0169-9

**Published:** 2016-12-07

**Authors:** Paulo de Oliveira Vasconcelos Filho, Miriam Regina de Souza, Paulo Eduardo Mangeon Elias, Ana Luiza D’Ávila Viana

**Affiliations:** 1 Department of Preventive Medicine, School of Medicine of University of São Paulo, Av. Dr Arnaldo, 455 2° andar, São Paulo, 01246-903 SP Brazil; 2 http://www2.fm.usp.br/preventiva; 3 435/21 R Estado de Israel, Sao Paulo, 04022-001 SP Brazil

**Keywords:** Physicians, Job satisfaction, Motivation, Academic medical centers

## Abstract

**Background:**

Physician shortage is a global issue that concerns Brazil’s authorities. The organizational structure and the environment of a medical institution can hide a low-quality life of a physician. This study examines the relationship between the hospital work environment and physicians’ job satisfaction and motivation when working in a large public academic hospital.

**Methods:**

The study was restricted to one large, multispecialty Brazil’s hospital. Six hundred hospital physicians were invited to participate by e-mail. A short version of the Physician Worklife Survey (PWS) was used to measure working satisfaction. Physicians were also asked for socio-demographic information, medical specialty, and the intention to continue working in the hospital.

**Results:**

Data from 141 questionnaires were included in the analyses. Forty-five physicians graduated from the hospital’s university, and they did not intend to leave the hospital under any circumstance (affective bond). The motivating factor for beginning the career at the hospital and to continue working there were the connection to the medical school and the hospital status as a “prestigious academic hospital”; the physicians were more satisfied with the career than the specialty. Only 30% completely agreed with the statement “If I had to start my career over again, I would choose my current specialty,” while 45% completely agreed with the statement “I am not well compensated given my training and experience.” The greater point of satisfaction was the relationship with physician colleagues. They are annoyed about the amount of calls they are requested to take and about how work encroaches on their personal time. No significant differences between medical specialties were found in the analysis.

**Conclusions:**

The participants were satisfied with their profession. The fact that they remained at the hospital was related to the academic environment, the relationship with colleagues, and the high prestige in which society holds the institution. The points of dissatisfaction were inadequate remuneration and the fact that work invaded personal time. Routinely, there is a need for organizations to examine the impact of their structures, policies, and procedures on the stress and quality of life of physicians.

## Background

Several structural changes have occurred in the medical profession over the past 40 years [1, 2]. Despite the exhaustive nature of the work, physicians used to have great autonomy, decided on their working conditions, and had good financial returns and security at work [3]. In all over the world, there has been a growing wave of dissatisfaction with the work itself [4–8].

The job satisfaction of physicians is in the interest of the entire healthcare context [9]. According to Locke [10], job satisfaction is defined as a pleasurable or positive emotional state, resulting from the appraisal of one’s job or job experiences. It is the result of complex interactions between the work experience and the organizational environment [10]. It results from a multidimensional construct, which incorporates both cognitive and affective elements [10, 11]. High physicians’ job satisfaction benefits their physical and mental health and well-being and relates negatively to burnout, intention to leave, and job or career turnover [12, 13]. It relates positively to quality of care and patient satisfaction, promoting more conscientious prescription behaviors, less self-reported suboptimal patient care practices, and less self-reported likelihood of making errors [9, 12, 13]. Sometimes, the positive influences of job satisfaction may imply economic value for society as a whole [12, 14]. Physicians who are depressed or burned out can be more apt to practice medical errors that cause injury or death as consequence [15].

Job satisfaction can be divided into extrinsic and intrinsic factors [10]. The extrinsic factors relate to the architecture of the job (pay, working conditions, and hours of work). The intrinsic factors relate to psychological attributes of the job (nature, ability, and recognition). Considering the importance of job satisfaction to physicians’ well-being and to quality of care, it is important to investigate factors that contribute to or decrease job satisfaction [9]. The factors that may cause satisfaction are different from those responsible for causing dissatisfaction; the two feelings cannot be treated as opposites of one another. The opposite of satisfaction is not dissatisfaction but “no satisfaction” [8].

Physicians’ dissatisfaction with the health system in which they operate has become a major problem in many countries [6, 15]. Dissatisfied physicians may be more likely to unionize, to strike, to experience medical problems themselves to reduce their working hours, seek other activities, and retire as early as possible [13–18]. Physician dissatisfaction is related to lower patient treatment adherence, deterioration in the quality of care received, prescription errors, and increased rates of medical errors, which jeopardize patient safety [17–19]. The causes of job dissatisfaction are multiple and complex. Various factors related to job dissatisfaction are listed in Table 1.

**Table 1:** Current causes of medical dissatisfaction

Extrinsic	Intrinsic
✓ Income reduction	✓ Loss of prestige and autonomy
✓ Work overload	✓ Conflicts between personal, family, and work life
✓ Time pressure	✓ Threat of malpractice proceedings
✓ Changes in the organization and administration of the working environment	✓ Depreciation between specialties
✓ Decrease in freedom imposed by types of procedural standards	✓ Lack of respect and appreciation of the work offered
✓ Reduced time for appointments, which leads to a worsening of the doctor-patient relationship	✓ Aggressiveness and violence from patients and the community

Extrinsic factors are potent sources of job dissatisfaction; however, improving such factors above a minimum acceptable level does not produce sustained improvements in job satisfaction. Attention to intrinsic job factors is more important for promoting high levels of job satisfaction [14, 15]. Satisfied physicians perform their role better, which has a positive impact on satisfaction and patient adherence [8].

Another aspect that managers always need to evaluate is motivation. It is intrinsically linked to satisfaction and involves cognitive, affective, and behavioral processes [19]. In the work context, motivation can be defined as an individual’s degree of willingness to exert and maintain an effort to achieve personal and organizational goals [20]. The presence of motivated physicians in a service leads to better performance and high levels of satisfaction among workers, even if other health service characteristics fall below the standards that are recommended for health institutions [21, 22]. Low remuneration only partly explains the low levels of motivation [23]. There are nonfinancial factors that seem to play a major role in the motivation of health workers. These factors include the availability of resources, opportunities for training and promotion, issues relating to supervision and management, and communication within the organization. Neither job satisfaction nor motivation is directly observable, but both have been identified as critical for the retention and performance of health workers [21, 22].

Having a qualified medical team, highly motivated, and with available resources can be the basis for improving results in healthcare provision. However, how can one ensure and evaluate these parameters? The evaluation of satisfaction in the workplace has been the method that many authors have used to verify professional retention by the health system [24–28]. More recently, some authors have performed studies about motivation; however, there is relatively little empirical evidence available on this issue, especially in low-income countries [22, 23].

It is therefore more likely that the global understanding of motivation is low, and physicians tend to follow the market behavior. They are attracted to work in private units or in developed countries. This is a common path for professionals with proper training from recognized institutions in developing countries [22]. When the physician’s training center is a public university supported by the government (society), the university is left with just the burden of training. Thus, society pays for and assists in this training, without receiving the due return on its investment [21, 29].

In the Brazilian unified health system, academic hospitals are at the top of the chain once they have succeeded in developing a larger structure. They have more resources and perform more complex treatments. However, similar to other players in the system, they can occasionally experience difficulties in financing and management. Most are linked to a public university [30]. Success in fulfilling the organizational missions, which are clinical excellence, pioneering research, and superior education and training, depends on the effectiveness of the entire workforce. If the workforce is engaged and aligned with these missions, then it will be a valuable and strategic resource to drive organizational performance [31].

The complexity and the environment of a medical institution can hide a low quality of life and the harmful effects associated therewith [32]. Highly complex medical services place a high demand on workers. The loss of well-trained physicians can compromise the system’s ability to provide adequate care to patients. Furthermore, in an academic medical center, a lack of balance in the specialty mix of physicians is inappropriate to maintain the quality of medical learning. High-paying specialties such as surgical subspecialties were more likely to be seduced by the private system as compared to lower-paid specialties such as psychiatry or internal medicine [17]. More experienced workers may leave the service because they have skills that are highly sought after. The decrease in the absolute number of physicians increases the workload and stress levels and demotivates those who have remained in the service [31–33].

This study examines the relationship between the hospital work environment and physicians’ job satisfaction and shows which are the motivations to continue working in a large public academic hospital.

## Methods

This was a cross-sectional study of physicians working at the Clinics Hospital, School of Medicine, University of São Paulo (CH-MS-USP), Brazil. The stages were the formulation of the research problem, data collection, data evaluation, data analysis, and presentation of results.

### Study location

The CH-MS-USP complex was chosen for this study because of its significance, size, and importance in treatment and research in Brazil and Latin America [34, 35]. It is one of the most important centers for the dissemination of technical and scientific information to the national health system. It belongs to the unified national health system (SUS-public) and must follow its guidelines [34, 36]. The hospital is highly complex and is subject to the advantages and difficulties of the system. The government funds the CH-MS-USP and is also responsible for medical remuneration [34, 36].

### Development phase

The literature review showed that the different medical services have developed their own methodologies of measuring satisfaction, using instruments that had been previously validated in the health literature. Even the World Health Organization offers some questionnaires for surveying the professional profile and degree of satisfaction [37].

In 2004, based on an instrument developed by Machado [38], the National Board of Medicine performed a national survey with 8980 physicians (3% of the total) with regard to qualifications, work, and quality of life [39]. This survey was conducted electronically. In addition to profiling the Brazilian physician, the study attempted to ascertain his/her level of job satisfaction. The number of low-paying jobs was remarkable. The majority of respondents considered their profession exhausting. Many had more than three work activities, and their workload had increased in recent years. The rise in workload did not bring a significant increase in income. The professionals appeared despondent and disillusioned with the traditional health system and had a pessimistic view of the future of their career.

This study followed the formula used in 1999 by Konrad et al. [26] in the United States of America; a version of the Physician Worklife Survey (PWS) questionnaire was used with the section on the local community removed to allow a greater focus on the working environment itself (Appendix 1). We translated the PWS to Portuguese and adapted the version for Brazil based on a study published by Machado [40]. To analyze its applicability, the instrument was initially sent electronically to CH-MS-USP physicians, and a further review of the responses was performed. At this stage, 100 questionnaires were sent, and 27 (27%) positive responses were received. Despite the fact that we had a higher percentage of responses that that national survey had, some responses were not even answered. As the number of participants was small, we decided to proceed with the study of larger scale.

### Sample

Through the electronic addresses of the institution’s physicians, a total of 600 letters of invitation to participate in the study were randomly sent to physicians of different areas who had been at the institution for more than 6 months. After accepting the terms of consent, they were able to complete the electronic questionnaire. A total of 180 physicians agreed to participate in the study. Questionnaires that were not fully completed were excluded from this total. The final sample comprised 141 physicians. The questionnaires were completed between August 2013 and July 2014.

### Instrument

The first section of the questionnaire was formed by socio-demographic data regarding age, gender, training, specialization, post-graduate qualifications, and the opinion about the place of work. In addition, we did three questions to know which were the crucial factors to begin and to continue working or to leave the hospital.

The second section of the questionnaire contained questions developed by the CSSG/SGIM studies, first published by Konrad et al. [26] and Williams et al. [27] and then by Linzer et al. [28]. This section contains many questions regarding working in health care, satisfaction with one’s career, and job satisfaction in the institution.

In total, there were 44 career and job satisfaction questions. A total of 10 satisfaction domains were measured: career, expertise, and autonomy; relationships with patients; relationships with colleagues; relationships with staff; personal time; income and resources; and the inherent characteristics of the work. Each item used a modified Likert response format, with a scale ranging from 1 to 5. The questions could be positive or negative in nature, as shown in Appendix 1.

The answers were provided on a unipolar scale of five points for each statement. A score of 1 represented “strongly disagree,” 2 “disagree,” 3 “neither agree nor disagree,” 4 “agree,” and 5 “strongly agree.” The advantages of this type of questionnaire in measuring overall job satisfaction are brevity, the possibility of validation, and increased sensitivity in measuring changes in job satisfaction [39, 40].

### Statistical analysis

To meet the study objectives, in addition to the basic techniques of exploratory data analysis such as the mean, median, standard deviation, minimum, maximum, and absolute and relative frequency, three other statistical analytical techniques were used: Cronbach’s alpha, factor analysis, and the Kruskal-Wallis test. Cronbach’s alpha was used to evaluate whether the instrument was reliable and capable of producing stable and consistent measurements. This coefficient was analyzed separately for “satisfaction with career and area of activity” and “job satisfaction.”

Factor analysis was used to ascertain the instrument’s latent factors and to establish which questions were the most important in each of these factors. This test is sensitive to the size of the sample, so it was applied the Kaiser-Meyer-Olkin, comparing the correlations with the partial correlations observed between variables. To produce a better interpretation of the factors, it is common practice to make a rotation or a transformation of the factors. The most common method of rotation is the orthogonal varimax method, which was performed, too [41]. Before the development of factor analysis, Bartlett’s test was applied. This test confirms the possibility and adequacy of the factor analysis method for the treatment of data by verifying whether there are desirable correlations between variables. Finally, the Kruskal-Wallis test was used to statistically evaluate the association of the latent factors obtained by factor analysis and each question on satisfaction.

### Approval

The project was reviewed and approved by the MS-USP Department of Preventive Medicine and the CH-MS-USP Research Project Analysis Ethics Committee under number 0176/11.

## Results

A total of 63% of the 141 physician respondents were male. A total of 70% were from the state of São Paulo, 32% received their degrees from FMUSP, 34% received their degrees from other public schools, and 34% received their degrees from private colleges. Most of the physicians (84%) do not have either master or doctorate degrees. A total of 77% were aware of their gross salary. Only 3.5% of the physicians considered the quality of the unit’s service as “Excellent,” and 54% evaluated the quality of the service as “Good”—adequate (Table 2).

**Table 2:** Descriptive analysis of the physician sample

Variable			Number	Percent
Gender	Female		52	37
	Male		89	63
School of medicine	MS-USP		45	32
	Public, not MS-USP		48	34
	Private		48	34
Brazilian regions	Northeast	AL (3) BA (2) MA (2) PB (1) PE (2)	10	7.1
	Midwest	DF (1) MS (1)	2	1.4
	North	GO (2) PA (1)	3	2.1
	Southeast	ES (1) MG (14) SP (99) RJ (2)	116	82.2
	South	PR (8) RS (2)	10	7.1
Place of residency	Hospital researched		96	68
	External		45	32
Degree titles	None		84	60
	Master		15	11
	Doctor		42	30
Physicians know how much they earn from the hospital	Yes		109	77
	No		32	23
How do you consider the quality of the facility	Excellent		5	3.5
	Very good		40	28.6
	Good		76	54
	Bad		20	14.3
Total			141	100

*MS-USP* Medical School of University of São Paulo, Brazilian States: *AL* Alagoas, *BA* Bahia, *MA* Maranhão, *PB* Paraíba, *PE* Pernambuco, *DF* Distrito Federal, *MS* Mato Grosso de Sul, *GO* Goiás, *PA* Pará, *ES* Espirito Santo, *MG* Minas Gerais, *SP* São Paulo, *RJ* Rio de Janeiro, *PR* Paraná, *RS* Rio Grande do Sul

Table 3 shows the quantitative variables addressed in the questionnaire. The mean age was 43.43 years (±10.56, with a minimum of 28 and maximum of 67). The mean treatment hours were 22.37 h/week (±14.31). The mean time for other patient-related activities was 4.15 h/week (±5.81). The mean time spent in meetings was 2.93 h/week (±3.19), and the mean time spent on other work-related activities was 3.88 h/week (±5.28).

**Table 3:** Mean, median, standard deviation, minimum and maximum age and hours worked by the physician sample

Variable	Mean	Median	SD	Min.	Max.
Age (years)	43.43	42	10.56	28	67
Medical care (h/w)	22.37	20	14.31	0	64
Other activities with the patient (h/w)	4.15	2	5.81	0	40
Meetings (h/w)	2.93	2	3.19	0	15
Other activities at work (h/w)	3.88	2	5.28	0	30

*h/w* hours/week, *SD* standard deviation

The number of questionnaires answered by each medical specialty is shown in Table 4.

**Table 4:** Frequency of respondents and the total number of physicians in each specialty

Specialty	Number	Percent	Physicians in each specialty	% of each specialty in the hospital
Pediatrics	20	14.1	151	8.4
Anesthesiology, cardiology	19/19	13.4/13.4	120/190	6.7/10.6
Medical clinic	17	12	297	16.6
General surgery	10	7.1	93	5.2
Infectious disease	6	4.2	45	2.5
Critical care medicine, geriatrics	5/5	3.5/3.5	60/33	3.3/1.8
Cardiovascular surgery, psychiatry	4 each	2.8	27/26	1.5/1.4
Pathology, pneumology, vascular surgery	3 each	2.1	21/30/19	1.0/1.3/0.5
Thoracic surgery, head and neck surgery, endocrinology, preventive, rehabilitation, radiology, rheumatology	2 each	1.4	25/10/40/23/18/42/23	1.3/0.5/2.2/1.2/1.0/2.3/1.2
Plastic surgery, GO, gastroenterology, forensic medicine, nephrology, neurology, orthopedics, and urology	1 each	0.7	15/44/55/14/25/37/58/45	0.8/2.4/3.0/0.7/1.3/2.0/3.2/2.5/11.1
Otorhinolaryngology, opthalmology, and others	0	–	200	11.2
Total	141	100	1786	100

Some factors were associated with the turnover (beginning, continuing, and leaving) of medical staff in the CH-MS-USP, as shown in Table 5. The factors that motivated the physician’s starting his/her career at the hospital were mainly the connection between the hospital and the medical school, which allowed continued contact with an academic environment (17.0%). The facility being a “prestigious teaching hospital” was the second reason for a physician to start his/her career at the hospital, with 22 replies (15.5%). The opportunity to develop studies or research was what motivates the physicians to stay at the institution (16%). Thirty-one said they never wanted to stop working at the hospital (22%). Those who would leave (78%) mainly said that it would be due to the worsening workload (22%).

**Table 5:** Physicians motivation factors of beginning, continuing, and leaving the Clinics Hospital, MS-USP

Question	Answer	Number	Percent
Which factor led to you beginning to work at the hospital?	Academic support	24	17
	Renowned academic hospital	22	15.6
	Specialty	16	11.4
	Research	12	85
	Others (invitation, public job benefits, training)	67	47.5
Which factor led you to stay working at the hospital?	Research	23	16.5
	Renowned academic hospital	19	13.5
	Clinical practice	17	12
	Academic support	17	12
	Others (post-graduation, training, public job benefits, retirement)	65	46
Which factor would lead you to resign?	I would never resign, in any case	31	22
	Worsening of the workload	31	22
	Worsening of the workplace	26	18.5
	Invitation from another institution	18	12.7
	Others (lack of interest, staff/boss exchange, academic career)	35	24.8
Total		141	100

*MS-USP* Medical School of University of São Paulo

About career and specialty, answers to the questions listed in Appendix 1, the statement “If I had to start my career over again, I would choose my current specialty” had the highest proportion of individuals who completely agreed (30%). On the other hand, the statement “If I could choose again, I would not be a physician” had the highest proportion of those who totally disagreed (50%), followed by disagree (28%), neutral (10%), agreed (6%), and totally agreed (5%).

Regarding working at the hospital, the statement with the highest proportion of respondents who completely agreed was “I am not well compensated given my training and experience” (45%). The statement with the highest proportion of respondents who strongly disagreed was “My current work situation is a major source of frustration” (40%).

Cronbach’s alpha was then calculated to evaluate the reliability of the instrument for questions on career and area of activity satisfaction and questions regarding job satisfaction. The overall Cronbach’s alpha in relation to career and area of activity satisfaction was 0.77. The overall Cronbach’s alpha in relation to job satisfaction was 0.82. These Cronbach’s alpha values allow us to conclude that the applied instrument is reliable and produces stable and consistent measurements (Table 6).

**Table 6:** Cronbach’s alpha, Kaiser-Meyer-Olkin measure, and Bartlett’s test of sphericity for job and career satisfaction

	Dimensions	Alpha	Kaiser-Meyer-Olkin	Approximated chi-square	Bartlett’s test	
Job satisfaction	Autonomy	0.82			*df*	*p* value*
	Personal time	0.84				
	Relationship with patients	0.82				
	Relationship with colleagues	0.82				
	Relationship with staff	0.81	.709	324 871	28	.000
	Income	0.83				
	Work	0.82				
	Resources	0.81				
	General	0.82				
Career satisfaction		0.77	.773	2 840 464	595	.000

*df* degrees of freedom

*Bartlett’s test *p* ≤ 0.05

Factor analysis was used to investigate the instrument’s latent factors and to establish which questions were the most important in each of these factors. Regarding career and specialty, the questions with the highest factor loadings were as follows: “In general, my medical career has met my expectations,” “My specialty no longer has the appeal to me it used to have,” and “In general, the practice in my specialty has met my expectations.”

Regarding job satisfaction at the hospital, the questions with the highest factor loadings were as follows: “My physician colleagues values my perspective in practice,” “I am overwhelmed by the needs of my patients,” and “Overall, I am satisfied in my current place of work.”

The Kruskal-Wallis test was then used to evaluate the association of the latent factors obtained by the factor analysis with the different specialties. No significant association was detected between the specialties and any of the detected latent factors. Thus, the orthogonal rotation varimax was performed on the entire sample. The results of KMO and Bartlett’s test are shown on Table 6.

Our results, after all tests, revealed statistical significance for the questions “The amount of call I am required to take is not excessive,” for answer “disagree”; “I get along well with my medical colleagues”, for answer “completely agree”; and “The work rarely encroaches on my personal time,” for answer completely disagree, with *p* values of 0.01, 0.03, and 0.008, respectively (Table 7).

**Table 7:** Mean, median, standard deviation, *p* value for following questions of job and career satisfaction

Dimension	Variable	Mean	Median	SD	*p*
Career and specialty satisfaction	If I were to choose over again, I would not become a physician.	0.98	1	0.91	0.62
	If I had to start my career over again, I would choose my current specialty.	3.63	4	1.28	0.83
	In general, my medical career has met my expectations.	3.62	4	1.07	0.88
	My specialty no longer has the appeal to me that it used to have.	3.11	3	1.39	0.58
	In general, the practice in my specialty has met my expectations.	3.71	4	1.13	0.77
Job satisfaction	I am not well compensated given my training and experience.	4.00	4	1.14	0.87
	My physician colleagues value my perspective in practice.	3.56	4	0.99	0.87
	My current work situation is a major source of frustration.	2.14	2	1.12	0.66
	I am overwhelmed by the needs of my patients.	2.82	3	1.18	0.56
	Overall, I am pleased with my work.	3.30	4	1.09	0.24
	The amount of calls I am required to take is not excessive.	2.86	3	1.24	0.01*
	I get along well with my physician colleagues.	3.98	4	0.91	0.03*
	The work rarely encroaches on my personal time.	2.32	2	1.19	0.008*

*SD* standard deviation

*Kruskal-Wallis test *p* ≤ 0.05

## Discussion

Our results showed that the physicians who participated in this study are satisfied with their careers and that most would not choose another profession if they could choose again. Regarding the job satisfaction, good relationship with professional colleagues was the greatest source of satisfaction. On the other hand, physicians believe they are not well paid for the activities they perform, and the work encroaches on their personal time (even though the median time spent at the institution does not exceed 26 h/week). More than 50% of the physicians consider the workplace as “good” (in the average). Considering that the hospital is one of the best public hospitals in the country, it was expected that the degree of quality perceived by them would be higher. Only five respondents consider the hospital excellent. The positive factors that cause professionals to continue working at the institution are the possibility of research development, the prestige in which society holds the hospital, and the connection with the school of medicine.

The study had limitations that could not be overcome. The number of refusals to participate was higher than that expected. If we had kept the same response rate as we achieved in the development phase, the sample would have been more representative. Even with reminders, it was not feasible to get the same rate of response. Other authors have also reported sampling problems [42–44]. Most of the physicians do not spend so many hours in the CH-MS-USP, so we opted to use an electronic questionnaire and a quantitative sample. Since it was a self-administered questionnaire, it is therefore possible that the respondents might have over- or under-reported their level of satisfaction. We cannot generalize the results to health professionals working in the private sector in the country. The satisfaction factors considered were mainly related to working at the institution. Our objective was to investigate the effect of a specific high complex medical service in physician’s satisfaction. It is known that health conditions and external or family changes can also affect workers’ satisfaction [42–45]. However, our interest was to verify the intrinsic factors connected to the institution in terms of satisfaction.

The evaluated hospital is part of the Brazilian unified health system. According to the system’s organization, it is a tertiary hospital that is at the top of the health chain, offering more resources, and performing more complex treatments. Similar to other members of the system, it occasionally experiences difficulties in financing and management [30, 36]. The hospital has a peculiar nature in that it is to be a health profession’s training center linked to a public university. It can sometimes retain healthcare professionals due to an emotional bond (their desire to work at a prestigious institution), despite the difficulties encountered by some professionals (as observed in the response: “I would not resign under any circumstances”).

As mentioned above, our results showed that good relationship with professional colleagues was the greatest source of satisfaction. Linzer et al. [28] and Lambrou et al. [45] also find that that factor tends to increase the job satisfaction. The physicians do not spend so many hours in the CH-MS-USP. It is probable because they are trying to increase the income having more than one job (though, this aspect was out of our scope). Also, 23% did not know their gross salaries. This characteristic may be related to the different sources of income. The CH-MS-USP remuneration is a regular state physician wage, which may be slightly supplemented according to position and length of service. The differences regarding remuneration were not evaluated. However, studies show that variables relating to the work itself may be more important than remuneration [46, 48].

Several factors affect a physician’s professional decision to leave patient care [42]. The process of losing physicians from public teaching hospitals typically begins after graduation. Among the publications studied [42, 43, 45–47, 49–51], there is no unanimity with regard to the factors that affect turnover, with aspects such as age, specialty, and post-graduation studies having been identified. However, there is a trend to agree that the more specialized physicians tend to remain in higher-complexity services. Our sample showed no change in satisfaction between the different specialties or between those who had or had not obtained post-graduate degrees. In relation to gender, no significant differences were detected in total job satisfaction scores between male and female medical staff. Similar results were found by Hills et al. [52].

Wai et al. [31] conduct a large survey of academic surgeons on the workplace factors that affect their satisfaction and intention to leave. They conclude that institutional understanding and the improvement of specific environmental work factors can improve the recruitment and retention of teachers [31]. As noted by Coleman in 2015, the variables associated with physicians’ motivation and satisfaction in staying in a health service are numerous, and aspects that generate dissatisfaction can be strong enough to cause the professional to leave the institution. Reasons that are only emotional and not managerial may be insufficient in maintaining a low level of turnover of human resources [53].

For Bezerra [54], most of the time, the insertion of the professional into the labor market occurs through an institution, where he/she becomes part of the staff and establishes work relationships. Variations in the frequency and durability of these bonds constitute elements of staff turnover in businesses. If the quantity of human resources is not maintained at adequate levels, then excessive movement can cause an imbalance in the workforce and consequently undesirable effects on the quality and quantity of the services provided [54].

In recent decades, physicians have faced an overload of occupational demands that had not been observed before. There are a larger number of patients to treat, greater administrative demands, and a need to constantly keep up to date regarding preventive and treatment methods [15, 27, 47, 53]. Dissatisfaction with the work process occurs when working day is long or when it affects family life [47, 53]. The greater the physician’s dissatisfaction is, the greater the tendency towards the increased turnover of these professionals in the service. In terms of medical treatment, this increase in turnover has consequences for patient care. There may be discontinuities in care, and if the quantity of physicians is insufficient, then those who remain will be overloaded. Patients may require additional treatment time with new physicians, until the latter are fully informed of each patient’s case. This places an unnecessary burden on the health service. Furthermore, turnover itself incurs costs for the service. An even worse effect of dissatisfaction is disinterest in providing care, which may generate greater dehumanization and an increased incidence of medical errors [47, 53, 55].

Other studies have also associated psychosocial work conditions with dissatisfaction with regard to the workplace. These include characteristics such as low quality of staff [16], little or no support from colleagues and superiors [55], high psychosocial stress (as measured by work stress and effort-reward imbalance) [56], constant changes in the individual’s position at the institution [46], and the low possibility of choosing one’s method of working [57]. We do not find these institutional characteristics in our sample.

The results of this study can be potentially important when human resource management policies are to be applied in this particular scenario. Hospital management should strive to evaluate and stimulate the factors that will lead to the motivation of the medical staff and combat factors that generate dissatisfaction [58].

## Conclusions

Although other studies have noted physicians’ growing dissatisfaction with their careers, in this survey, the professionals who participated in the survey appeared satisfied with their choice of profession. The fact that they started and remained at the hospital was related to the academic environment and the high prestige in which society holds the institution. The main satisfaction factor in this study was the physician’s relationship with colleagues. Job satisfaction is crucial for the stability of any medical service.

The points of dissatisfaction that were found were inadequate remuneration and the fact that the work invaded personal time. It should be taken into account that these aspects may not only be related to work in the evaluated hospital because the time dedicated to work is not unique to this institution. Routinely, there is a need for organizations to examine the impact of their structures, policies, and procedures on the stress and quality of life of physicians.

Public health services should implement people management strategies to protect their workers and recognize that professionals are autonomous individuals with rights. A growing number of studies are exploring the connection between incentives/motivation, satisfaction, and worker retention [59]. Human resource managers should conduct systematic reviews to assist in the formulation of policies to manage physician dissatisfaction [60]. Given that reasons for dissatisfaction vary, they must be combated preventively. Otherwise, the loss of professionals may become an inconvenience for the institution.

## Appendix 1

**Table 8:** Items on satisfaction measure

	Statements	Score
Autonomy	Protocols and clinical guidelines restrict my freedom to practice.	−
	Outside reviewers rarely question my professional judgment.	+
	Formularies or prescription limits restrict the quality of the care I provide.	−
	I am able to refer patients or receive referrals when necessary.	+
	Gatekeeping requirements seldom conflict with my clinical judgments.	+
Personal time	Work rarely encroaches on my personal time.	+
	Interruption of my personal life by work is a problem.	−
	The number of calls I am required to take is not excessive.	+
Relationship with patients	I feel a strong personal connection with my patients.	+
	The gratitude displayed by my patients keeps me going.	+
	I am overwhelmed by the needs of my patients.	−
	Many patients demand potentially unnecessary treatments.	−
	Time pressures keep me from developing good patient relationships.	−
	Many times, I feel like what I do for my patients is just a drop in the ocean.	−
Relationship with colleagues	My physician colleagues are a good source of professional stimulation.	+
	I get along well with my physician colleagues.	+
	My physician colleagues value my perspective in practice.	+
	My physician colleagues are an important source of professional support.	+
	It is easy to communicate with physicians with whom I share patients.	+
Relationship with staff	The nonphysician staff where I work respect my professional judgment.	+
	My nonphysician colleagues are a source of personal support.	+
	The nonphysician staff in my practice is not accommodating.	−
	The nonphysicians in my practice reliably carry out clinical instructions.	+
Income	My salary is fair.	+
	I am not well compensated given my training and experience.	−
Job satisfaction	I have too much administrative work to do.	−
	Overall, I am pleased with my work.	+
	My current work situation is a major source of frustration.	−
	My work in this practice has not met my expectations.	−
Resources	Overall, I am satisfied with my current place of work.	+
	I have sufficient exam room space to see my patients.	+
	Medical supplies are available when I need them.	+
Career satisfaction	If I were to choose again, I would not become a physician.	−
	All things considered, I am satisfied with my career as a physician.	+
	In general, my medical career has met my expectations.	+
	I would recommend medicine to others as a career.	+
	I feel rewarded by my performance in the clinic at the moment.	+
Specialty satisfaction	My specialty no longer has the appeal to me it used to have.	−
	In general, the practice in my specialty has met my expectations.	+
	If I had to start my career over again, I would choose my current specialty.	+
	My specialty has not provided more job stability than I used to have.	−

## Abbreviations

CH-MS-USP: Clinics Hospital, Scholl of Medicine of University of São Paulo
CSSG: Career Satisfaction Study Group (USA)
MS-USP: Medical School of University of São Paulo
PWS: Physician Worklife Survey
SD: Standard deviation
SGIM: Society of General Internal Medicine (USA)
SUS: Brazil’s unified national health system
USA: United States of America
